# A Model for Integrated Behavioral Health Teams in Primary Care to Support Cognitive Screening for Older Adults

**DOI:** 10.1093/geroni/igaf122.4052

**Published:** 2025-12-31

**Authors:** Louisa Thompson, Stephanie Czech, Molly Lawrence, Charles Eaton

**Affiliations:** Alpert Medical School at Brown University, Providence, Rhode Island, United States; Alpert Medical School at Brown University, Pawtucket, Rhode Island, United States; Alpert Medical School at Brown University, Providence, Rhode Island, United States; Alpert Medical School at Brown University, Providence, Rhode Island, United States

## Abstract

Earlier detection of cognitive impairment among older adults is a growing clinical and public health priority, particularly as new interventions (both medical and behavioral) become available to manage mild cognitive impairment (MCI) due to AD. However, cognitive screening tools remain under-utilized in primary care due to barriers such as time, training deficits, and insufficient supporting research. Integrated Behavioral Health (IBH) teams employed in primary care settings are uniquely positioned to support care for older adult patients with MCI or dementia and their families. However, these teams often receive inadequate and/or inconsistent training in cognitive screening, brain health education, and caregiver support for this population. Further, Community Health Workers on IBH teams are often not equipped to screen or engage patients in care. We will present findings from a pilot study examining the feasibility and acceptability of an IBH-integrated tablet-based cognitive screening protocol. First, we will describe the approach to promoting awareness, training, and workflows for PCPs and IBH clinicians implementing the protocol. Second, we will share feasibility and acceptability findings from protocol implementation. During a 3-month period, 4 participating PCPs attempted 11 warm handoffs to the IBH team for cognitive screening. Thus far handoffs have been most successful during Medicare Annual Wellness Visits (83.3% completion rate) as compared to other routine follow-ups (40% completion rate). Finally, we will share lessons learned from iterative protocol development, examples of training materials, and case studies of successful and unsuccessful warm handoffs.

